# The Impact of Future Fuel Consumption on Regional Air Quality in Southeast Asia

**DOI:** 10.1038/s41598-019-39131-3

**Published:** 2019-02-25

**Authors:** Hsiang-He Lee, Oussama Iraqui, Chien Wang

**Affiliations:** 1 Center for Environmental Sensing and Modeling, Singapore-MIT Alliance for Research and Technology, Singapore, Singapore; 2 Energy and Environmental Engineering Department, National Institute of Applied Science of Lyon (INSA Lyon), Villeurbanne, France; 3 Center for Global Change Science, Massachusetts Institute of Technology, Cambridge, MA USA; 4 Present Address: Laboratoire d’Aerologie/CNRS/University of Toulouse, Toulouse, France

## Abstract

Aerosols emitted from fossil fuel burning can cause air quality and human health issues. In this sensitivity study, we examine the impact of fossil fuel aerosols on air quality in Southeast Asia under five different hypothetical fuel consumption scenarios. These scenarios reflect air pollutant outcomes of implementations of certain idealized policies in the power generation, industry, and residential sectors. Analyses based on comparison among the modeling results from these scenarios reveal the sectors that should be targeted by air pollution mitigation policy. The results reveal that in Southeast Asia, sulfate could be decreased by 25% if coal were to be replaced by natural gas in the power generation and industry sectors. Black carbon concentration would reduce 42% overall if biofuel were replaced by natural gas in the residential sector. Shipping emissions are especially critical for the urban air quality in Singapore: fine particular matters (PM_2.5_) could be dramatically cut by 69% in Singapore by merely eliminating shipping emissions.

## Introduction

Since the industrial revolution, fossil-fuel consumption has been systematically increasing worldwide. In recent years, the rise of energy demands in Southeast Asian countries is higher than most developed counties due to increasing population and fast-growing economies^1^. Such expansion in energy use causes an increase in fossil fuel consumption and associated emissions of pollutants, resulting in air quality and human health issues in the region^2–4^. In our previous study, fossil fuel burning aerosols were found to be responsible for the occurrence of 82% of the low visibility days (visibility <10 km), whereas biomass burning aerosols only contributed to 18% of the low visibility days in Southeast Asia^5^. Our result thus suggests that it is necessary to drastically reduce fossil fuel emissions in order to improve the air quality in Southeast Asia.

The awareness of climate change and associated problems has been gradually increasing within the general public and governments globally, resulting in the commitment of most countries to cut their emissions to a certain level (e.g., the Paris Agreement). Southeast Asian countries have also shown a strong willingness to work together on energy issues and to tackle environmental problems within the Association of Southeast Asian Nations (ASEAN). With a rapid economic growth of 5% from 2000 to 2013^1^ and an estimated increase in the population from 525 million in 2000 to 728 million in 2030^6^, Southeast Asian countries will have to face the challenge of increasing their energy consumption without increasing emissions. However, according to the International Energy Agency (IEA), with the current policies in the region regarding fuel consumptions, the energy demands of Southeast Asian countries would have to be mainly satisfied by burning fossil fuels, in particular coal, which is likely to become the primary fossil fuel in the energy mix of the region by 2040^1^. Therefore, ASEAN countries will need a change in their energy policies in order to be consistent with their environmental commitments.

In addition to coal usage, shipping is also an important emission source in Southeast Asia. Even in 20 years ago, annual emissions of sulfur dioxide (SO_2_) from international shipping in Asian waters were already estimated to be 0.236 Tg, representing about 11.7% of emissions in Southeast Asia^7^. In a recent study, Johansson *et al*.^8^ used Automatic Identification System (AIS) ship data for the year 2015 to investigate the global emissions from shipping. Their study shows that Singapore has the highest density of shipping emissions in the world. Besides that, the highest PM_2.5_ and SO_x_ emissions per unit area occurred in the eastern and southern South China Sea as well as over waters close to Southeast and South Asia. Ship-emitted nitrogen oxides (NO_x_) also enhances ozone (O_3_) production, leading to adverse effects on both human health^9^ and agricultural production^10^. However, despite a growing concern about the impact of atmospheric pollutants from ships on air quality in many ASEAN coastal cities, the regulation of air pollution in marine transport in the region remains unclear.

In fact, the impacts of current or alternative fuel consumption practices in the region on air quality have never been quantitatively analyzed, likely due to the complexity of such a task that requires the use of sophisticated weather and atmospheric chemistry models supported by emissions and measurement data.

Here we conduct a sensitivity study to examine the air quality outcomes over Southeast Asia in two selected years with sufficient data support, as results of five different hypothetic scenarios of fuel consumptions in the power generation, industry, and residential sectors. By exploiting a state-of-the-science regional weather and atmospheric chemistry model to simulate atmospheric concentrations of atmospheric pollutants including particulate matter, we examine the effects of these idealized emission-mitigation policies in altering atmospheric levels of several key air pollutants. In the paper, we first describe methodologies adopted in the study, followed by the results and findings from our assessment of the different hypothetical fuel consumption scenarios on the degradation of air quality over Southeast Asia. We then discuss the impact of trans-boundary pollution from surrounding countries on the air quality in Southeast Asia under these different scenarios in the discussion section. The last section summarizes and concludes our work.

## Methodology

### Model and emission inventories

**Figure 1:**
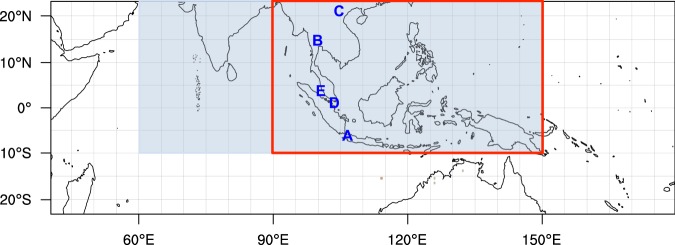
Model domain used for simulations. Blue color region indicates REAS fossil fuel emission coverage and the rest of domain uses EDGAR fossil fuel emission. The red box is the region of emission modification in Southeast Asia under different energy use scenarios (referred to as the SEA domain). A, B, C, D, and E indicate the location of five selected cities: Jakarta (**A**), Bangkok (**B**), Hanoi (**C**), Singapore (**D**), and Kuala Lumpur (**E**). This map is generated by the NCAR Command Language (Version 6.4.0) [Software]. (2017). Boulder, Colorado: UCAR/NCAR/CISL/TDD. (http://dx.doi.org/10.5065/D6WD3XH5).

The Weather Research and Forecasting model coupled with a chemistry module (WRF-Chem) version 3.6.1 is used in this study. We choose the Regional Acid Deposition Model, version 2 (RADM2) photochemical mechanism^11^ coupled with the Modal Aerosol Dynamics Model for Europe (MADE) and the Secondary Organic Aerosol Model (SORGAM)^12,13^ to simulate atmospheric chemistry and anthropogenic aerosol evolutions. The domain, as shown in Fig. 1, is designed to have a horizontal resolution of 36 km and 31 vertically staggered layers based on a terrain-following pressure coordinate system, and to include the tropical Indian Ocean on the west side in order to capture the path of Madden-Julian Oscillation (MJO), and to have a northern boundary confined within 23°N in latitude to avoid potential numerical instability from the terrain of Tibetan Plateau. The National Center for Environment Prediction FiNaL (NCEP-FNL) reanalysis data^14^ are selected for providing initial and boundary meteorological conditions, and for performing four-dimensional data assimilation (FDDA) to nudge model temperature, water vapor, and zonal and meridional wind speeds above the planetary boundary layer (PBL). The Mellor-Yamada-Nakanishi-Niino level 2.5 (MYNN)^15^ is chosen as the planetary boundary scheme in this study. The physics schemes in the simulations include Morrison (2 moments) microphysics scheme^16^, RRTMG longwave and shortwave radiation schemes^17,18^, unified Noah land-surface scheme^19^, and Grell-Freitas ensemble cumulus scheme^20^. The simulation periods are 2006 and 2008, representing a high year and an average year regarding precipitation, respectively (Fig. S1). The simulation of each year starts on 1 November of the previous year and runs for 14 months. The first two months are used for spin-up.

We notice that the default chemical profile in the lateral boundary condition is higher than the background concentration in our study region, and thus equivalent to provide additional aerosol sources from boundaries. Hence, we have set NO, NO_2_, SO_2_ and all primary aerosol levels to zero in the lateral boundary condition. We have also adjusted the ozone profile as shown in Fig. S2. The surface background mixing ratio of ozone from the World Meteorological Organization (WMO) Global Atmosphere Watch (GAW) station in Bukit Kototabang, Indonesia is 13.4 ppbv. We have thus adopted this value as the initial surface ozone level in the model and also scaled the tropospheric profile of ozone based on this new surface value.

Regional Emission inventory in ASia (REAS) version 2.1^21^ is a regional emission inventory for Asia including most primary air pollutants and greenhouse gases, and covering each month from 2000 to 2008. Due to the limited spatial coverage of REAS, we have used Emissions Database for Global Atmospheric Research (EDGAR) version 4.2 (2005 emission) (http://edgar.jrc.ec.europa.eu) for regions that are not covered by REAS within the domain (Fig. 1). In our pervious work^5^, we have compared the modeled results using REAS versus EDGAR emission inventories in a set of 1-year paired simulations. The differences between these two model runs are rather limited regarding aerosol-related variables.

### The design of fossil fuel emissions for various scenarios

Based on the current fossil fuel consumption and associated emissions in Southeast Asia, we have designed four policy scenarios and a reference scenario in the study. These scenarios are described in the following paragraphs.

*The Reference Scenario* is based on the original REAS fossil fuel emissions and complementary EDGAR emissions. This scenario provides a reference case for the real atmospheric concentrations of various pollutants that are not influenced by any hypothetical fossil fuel consumption changes during the period of simulation, and for analyzing the outcome associated with four other scenarios.

**Figure 2:**
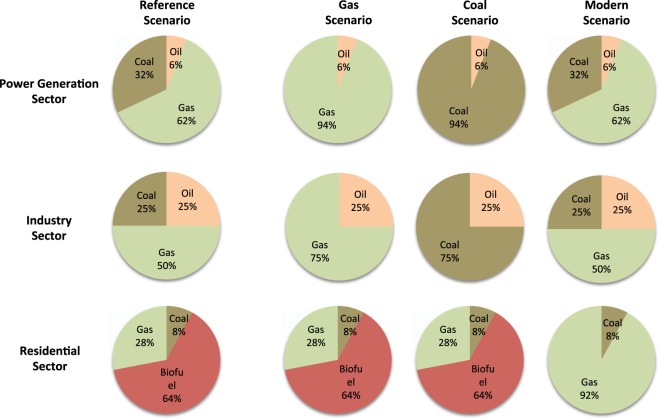
The first column shows the percentage of coal, oil, gas, and biofuel combustion fuels in the Power Generation, Industry and Residential Sectors in the Reference Scenario. From second to fourth columns are the percentage of each combustion fuel in the Gas, Coal, and Modern Scenarios, respectively. This figure is generated by the Microsoft PowerPoint (Version 14.7.2).

*The Gas Scenario* and *the Coal Scenario:* the former intends to mimic the implementation of a policy favoring the usage of natural gas in power generation and industrial sectors, while the latter intends to represent an opposite approach that is to enhance coal usage in these sectors. Specifically, in design, we assume that in the Gas Scenario, coal is no longer used in industry and power generation. Instead, all the energy produced by coal in these two sectors is replaced by the same amount of energy coming from burning of natural gas. Whereas in the Coal Scenario, we assume that natural gas is no longer used in industry and power generation, and all the energy produced in these two sectors comes from coal and existing oil burning (Fig. 2).

*The Modern Scenario* is designed to highlight the impact of policies that would favor the usage of gas and electricity in the residential sector (e.g., for cooking and heating) instead of the traditional biofuels. In this scenario, we assume that all biofuel energy of the residential sector is replaced by energy coming from natural gas rather than biofuel (Fig. 2).

Note that the emission modification domain in the Gas Scenario, Coal Scenario, and Modern Scenario is limited to the Southeast Asia region (10°S–23°N in latitude, 90–150°E in longitude; the red box in Fig. 1). The emission modification domain is referred to as the SEA domain in this paper. For convenience, the SEA domain includes a part of Southern China.

*The Shipping Scenario* is designed to highlight the contribution of pollutants emitted from marine traffic to worsening of regional air quality in Southeast Asia. We remove all aerosol and gas emissions from the shipping sector within the model domain to compare with the Reference Scenario.

The relevant calculations of all the above scenarios are done based on data of IEA^1^, and the fuel usages in each emission sector for various scenarios are shown in Fig. 2. Note that road transportation is an important pollution source in many major cities in Southeast Asia. However, due to the difficulty in obtaining the emission data that could represent the fuel usage, road conditions and distributions for Southeast Asia, we choose to focus on the above-described sectors in our study. Due to the availability of emission inventories, our simulations are limited to the year 2006 and 2008; therefore, the results of these simulations might not fully represent most current atmospheric concentrations. Nevertheless, such a selection in designing our simulations should not affect the relative changes of each hypothetical fossil fuel consumption in relate to the Reference Scenario.

Designing each one of these scenarios requires the modification of emissions in order to fit the assumption of each scenario. The emissions of five pollutants in the fossil fuel emission inventory – black carbon (BC), organic carbon (OC), nitrogen oxides (NO_x_), carbon monoxide (CO), and sulfur dioxide (SO_2_) – are adjusted by using different coefficients for the selected fuel in each sector. These coefficients are calculated using the share of each fuel in the energy sector given by IEA^1^ and also using emission factors from Ohara, *et al*.^22^.

**Figure 3:**
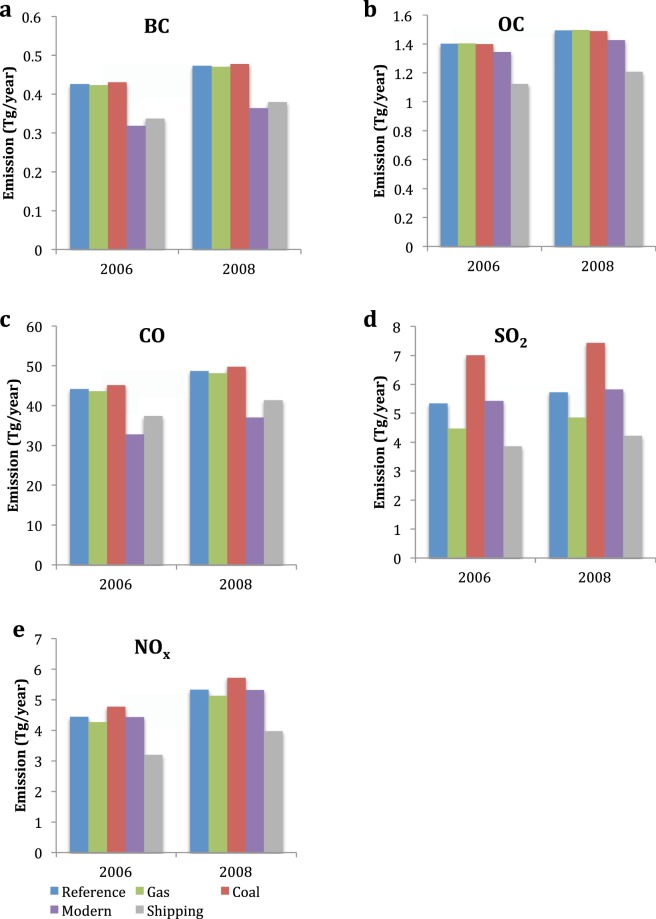
(**a**–**e**) Monthly mean BC, OC, CO, SO_2_ and NO_x_ emission in Reference, Gas, Coal, Modern and Shipping Scenarios averaged over 2006 and 2008, respectively. The data are calculated within the entire model domain but the emission changes in Gas, Coal and Modern Scenarios only cover the Southeast Asia region (the red box in Fig. 1). These figures are generated by the Microsoft Excel (Version 14.7.2).

Equation (1) is an example of the calculation of the coefficient related to SO_2_ emissions in the power generation sector for Gas Scenario.

1 $$c=\frac{{m}_{SO2}-\mathop{\overbrace{(\frac{{m}_{SO2}}{E{F}_{PG.REGIONAL}}\times { \% }_{PG.COAL}\times E{F}_{PG.COAL})}}\limits^{T1}+\mathop{\overbrace{(\frac{{m}_{SO2}}{E{F}_{PG.REGIONAL}}\times { \% }_{PG.COAL}\times E{F}_{PG.GAS})}}\limits^{T2}}{{m}_{SO2}},$$

where T1 is the total mass of SO_2_ that is emitted by coal power plants, and T2 is the mass of SO_2_ that would be emitted if the energy produced by coal in power generation were produced by natural gas. Indeed, we first divide the mass of SO_2_ $({m}_{SO2})$ by the average emission factor of power generation $(E{F}_{PG.REGIONAL})$ in order to get the energy used in power plants. We then multiply by the share of coal in power generation sector $({ \% }_{PG.COAL})$ (Fig. 2) and the energy factor of coal in power generation $(E{F}_{PG.COAL})$ (Table 1). The coefficients for the five pollutants in each one of the three sectors are presented in Table 2. Figure 3 shows the monthly BC, OC, CO, SO_2_, and NO_x_ emissions in 2006 and 2008 in five scenarios. Compared with 2006, emissions of all species have been increased in 2008.

**Table 1:** The energy factor of coal, oil, gas, and biofuel in the power generation, industry and residential emission sectors in the REAS emission inventory.

	SO_2_	BC	OC	NO_x_	CO
**Power Plant**					
Coal	504.2	1.2	0.3	267	154.3
Oil	674.4	8.1	6.1	303.1	83
Gas	8.6	0.4	0.3	189.8	319.1
**Industry**					
Coal	536.5	4.8	1.2	240.7	3934.3
Oil	310.3	3.2	2.4	81.2	44.3
Gas	41.8	3.2	15.7	79.7	2296.7
**Residential**					
Coal	376.4	147	119.3	122.9	5851.6
Biofuel	47.9	83.8	418.6	81.4	7467.2
Gas	124.2	2.4	4.8	74.1	102.7

**Table 2:** The Coefficients used to modify the emission sector of residential (Res), power generation (P.G.) and industry (Ind) in the REAS emission inventory for Gas, Coal and Modern Scenarios.

Scenario	BC			OC			SO_2_			NO_x_			CO		
	Res	P.G.	Ind	Res	P.G.	Ind	Res	P.G.	Ind	Res	P.G.	Ind	Res	P.G.	Ind
Gas	1	0.77	0.88	1	1	1.42	1	0.23	0.46	1	0.88	0.66	1	1.2	0.8
Coal	1	1.44	1.22	1	1	0.17	1	2.48	2.05	1	1.21	1.66	1	0.59	1.37
Modern	0.21	1	1	0.05	1	1	1.51	1	1	0.94	1	1	0.1	1	1

We also have designed another pair of simulations of the Gas, Coal and Modern Scenarios, which extend the emission modification region from the SEA domain to the whole model domain including a very large part of South Asia (referred to as *_ALL* in Sect. 4). The purpose of this design is to investigate whether air quality in Southeast Asia would be further affected by transboundary transport of pollutants if the different hypothetical fuel consumption policies were applied to other counties outside of Southeast Asia, especially India. The emission modification outside of the SEA domain also uses the same coefficients shown in Table 2.

## Results

### Model evaluation

Meteorological conditions, particularly wind fields and precipitation, could substantially influence the life cycle and transport path of aerosols. Hence, a rainfall comparison between observation and modeled results is conducted to evaluate the model’s performance in simulating meteorological features. The WRF simulation driven by NCEP-FNL reanalysis data produced a time series of monthly mean precipitation over 2006 and 2008 that closely followed that of satellite-retrievals from the Tropical Rainfall Measuring Mission (TRMM) 3B43 (V7) dataset^23^ despite a systematic overestimate (6.62 ± 0.48 mm day^−1^ versus 4.67 ± 0.32 mm day^−1^). Based on the sensitivity tests for FDDA grid nudging, the wet bias in the model results mainly comes from water vapor nudging. In our previous study^24^, we demonstrated a long term precipitation comparison with TRMM data using the same domain configuration but different aerosol emissions. The comparison showed that despite the model overestimate in average total precipitation, the temporal and spatial correlation of monthly rainfall between TRMM and modeled results are 0.68 and 0.86, respectively.

To evaluate the model’s performance in simulating the atmospheric abundance of several key pollutants, we have used surface PM_10_ concentrations derived from the Air Quality Index (AQI) in Kuala Lumpur, Malaysia and the observed CO and O_3_ levels from the World Meteorological Organization (WMO) Global Atmosphere Watch (GAW) station in Bukit Kototabang, which is located on the island of Sumatra, Indonesia. Note that in this study, we exclude biomass burning emissions to focus on the chemical concentration differences between each fuel consumption scenario. Therefore, we have also included in this comparison the results from our previous study^5^, where the same model configuration was used, but with both fossil fuel and biomass burning emissions included, referred to as FFBB.

The results show that the model simulated time evolution of PM_10_ is very close to that of observations, including peaks during the fire seasons (Fig. S3a). In comparison, as expected, the Reference Scenario results are very similar to those of FFBB except during the fire seasons. The background PM_10_ concentration produces by both FFBB and Reference Scenario, however, exhibits a systematic negative bias of 20 μg m^−3^ from the observations. As discussed previously in Lee *et al*.^5^, this discrepancy is likely due to the relatively coarse resolution of the model or an underestimation of aerosol and aerosol precursor emissions, or both. In Lee *et al*.^5^, we referred to Philip *et al*.^25^ study and highlighted that most global emission inventories do not include anthropogenic fugitive, combustion, and industrial dust (AFCID) from urban sources, e.g., fly ash from coal combustion and industrial processes such as iron, steel production and cement production, resuspension from paved and unpaved roads, mining, quarrying, and agricultural operations, and road-residential–commercial construction. In their study, they estimated a 2–16 μg m^−3^ increase in fine particulate matter (PM_2.5_) concentration across East and South Asia simply by including AFCID emission. A bigger gap for PM_10_ concentration is expected. Overall, the temporal correlation of daily PM_10_ between modeled PM_10_ in FFBB and observations is 0.55.

**Figure 4:**
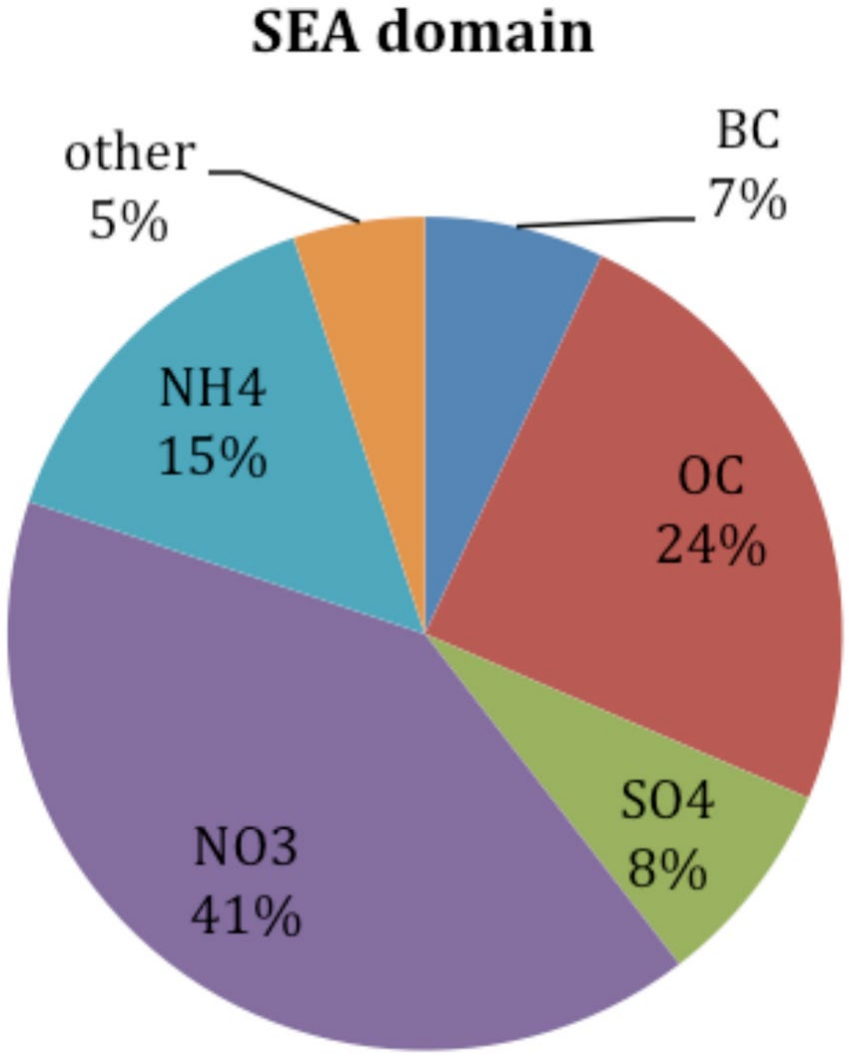
Pie chart of the mean chemical components of PM_2.5_ in Reference Scenario averaged over 2006 and 2008 in the SEA domain (the red box in Fig. 1). This figure is generated by the Microsoft Excel (Version 14.7.2).

For gaseous species, the model has accurately captured observed CO including both peaks and background levels (Fig. S3b). Since the primary source of CO in Bukit Kototabang is from biomass burning, all high CO levels occurred during fire seasons as reflected in FFBB results. In comparison, the Reference Scenario well captured CO evolutions during all the other periods. Additionally, the model has successfully simulated the evolution of surface O_3_ levels during the two simulated years. Nevertheless, the model indeed produced a positive bias of about 13 ppbv between observations (12.8 ± 5.1 ppbv) and the Reference Scenario (26.1 ± 5.1 ppbv), as well as about 23 ppbv between observations and FFBB (35.3 ± 11.0 ppbv) (Fig. S3c). The correlations for FFBB to observations in O_3_ and CO are 0.61 and 0.52, respectively. We notice that NO_x_ emission is higher in REAS emission inventory compared with several other emission inventories and estimates^21^. This could lead to the overestimate of background ozone in the model. High NO_x_ emissions in the REAS emission inventory could also have caused an overestimate of nitrate (NO_3_) in our simulation (Fig. 4) as suggested by the filter samples from the Surface PARTiculate mAtter Network (SPARTAN; http://spartan-network.weebly.com/), which show that the ratio of nitrate in PM_2.5_ concentration is much lower than that of sulfate (SO_4_) at all observational sites within the domain including Hanoi (Vietnam), Singapore (Singapore), Bandung (Indonesia), and Manila (Philippines) (Fig. S4). Observations from SPARTAN also indicate that there are missing anthropogenic aerosol components, mainly organic matter^25,26^, in the emission inventory. This could largely explain the underestimate of PM_10_ by the model.

### Spatial distributions and relative changes of indicative pollutants under different fuel consumption scenarios

**Figure 5:**
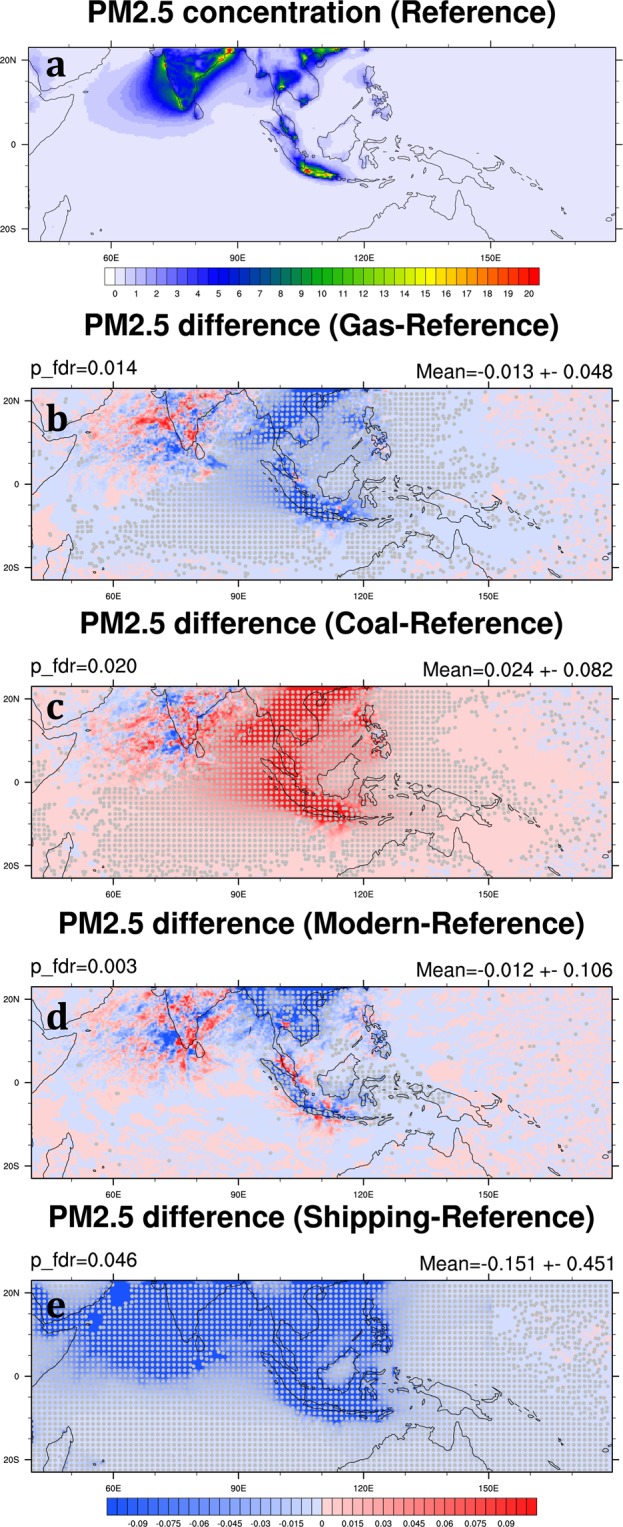
(**a**) The monthly mean PM_2.5_ concentration (μg m^−3^) in the Reference Scenario averaged over 2006 and 2008. (**b–d**) The PM_2.5_ concentration change (μg m^−3^) of the Gas, Coal, Modern and Shipping Scenarios to the Reference Scenario, respectively. Gray dots indicate differences that are statistically significant at a significance level of α_fdr_ = 0.05 after controlling the false discovery rate (FDR)^32,33^. The two-tailed p values are generated by Welch’s t test, using monthly mean data as the input. The approximate p value threshold, p_fdr, and area mean and standard deviation are written in above the map. These maps are generated by the NCAR Command Language (Version 6.4.0) [Software]. (2017). Boulder, Colorado: UCAR/NCAR/CISL/TDD. (http://dx.doi.org/10.5065/D6WD3XH5).

Fine particular matter (PM_2.5_) is one of the target air pollutants in our analyses because long-term exposure to PM_2.5_ may lead to chronic obstructive pulmonary disease, ischemic heart disease, lung cancer, and stroke^27,28^. In the Reference Scenario, high PM_2.5_ concentrations are found in major cities across Southeast Asia as well as India, including Hanoi, Bangkok, Jakarta, Kolkata, and Mumbai (Fig. 5a). The monthly mean PM_2.5_ in the SEA domain is 0.79 ± 0.13 μg m^−3^ averaged over 2006 and 2008 (Table 3). In general, the model underestimates PM_2.5_ concentration due to the 36-km model resolution used in this study and the missing anthropogenic fugitive, combustion and industrial dust emissions in the emission inventory^5,25^. The modeled chemical components of PM_2.5_ in the SEA domain suggest that nitrate aerosol is the major component of PM_2.5_ particles in Southeast Asia (Fig. 4). As mentioned above, it could come from high NO_x_ emissions in the REAS emission inventory. Under different hypothetical scenarios of fuel consumption in the SEA domain, the changes of PM_2.5_ concentration mainly occur locally (Fig. 5b–d); however, removing shipping emissions in the study domain reduces PM_2.5_ concentration significantly (Fig. 5e). Since chemical species come from various emission sectors, the spatial and temporal change patterns of these species also vary under different fuel consumption scenarios. Here, we focus on the changes of major PM_2.5_ components under different hypothetical scenarios.

**Table 3:** Mean and standard deviation of monthly mean concentration of PM_2.5_, BC, OC, SO_4_, and NO_3_ and mixing ratio of SO_2_, CO, and O_3_ in the SEA domain (10°S–23°N in latitude, 90°E–150°E in longitude) in each scenario averaged over 2006 and 2008.

	PM_2.5_ (μg m^−3^)	BC (μg m^−3^)	OC (μg m^−3^)	SO_4_ (μg m^−3^)	NO_3_ (μg m^−3^)	SO_2_ (ppbv)	CO (ppbv)	O_3_ (ppbv)
Reference	0.79 ± 0.13	0.05 ± 0.00	0.19 ± 0.01	0.06 ± 0.02	0.32 ± 0.08	0.09 ± 0.01	83.1 ± 1.3	23.3 ± 0.9
Gas	0.74 ± 0.13	0.05 ± 0.00	0.19 ± 0.01	0.04 ± 0.01	0.31 ± 0.09	0.06 ± 0.00	83.0 ± 1.2	23.2 ± 0.9
Coal	0.88 ± 0.15	0.06 ± 0.00	0.19 ± 0.01	0.11 ± 0.03	0.34 ± 0.09	0.16 ± 0.01	83.4 ± 1.3	23.3 ± 0.9
Modern	0.75 ± 0.13	0.03 ± 0.00	0.17 ± 0.01	0.07 ± 0.02	0.32 ± 0.08	0.10 ± 0.01	80.6 ± 1.1	23.3 ± 0.9
Shipping	0.58 ± 0.10	0.04 ± 0.00	0.15 ± 0.01	0.05 ± 0.01	0.22 ± 0.06	0.07 ± 0.01	82.0 ± 1.3	20.3 ± 0.7

**Figure 6:**
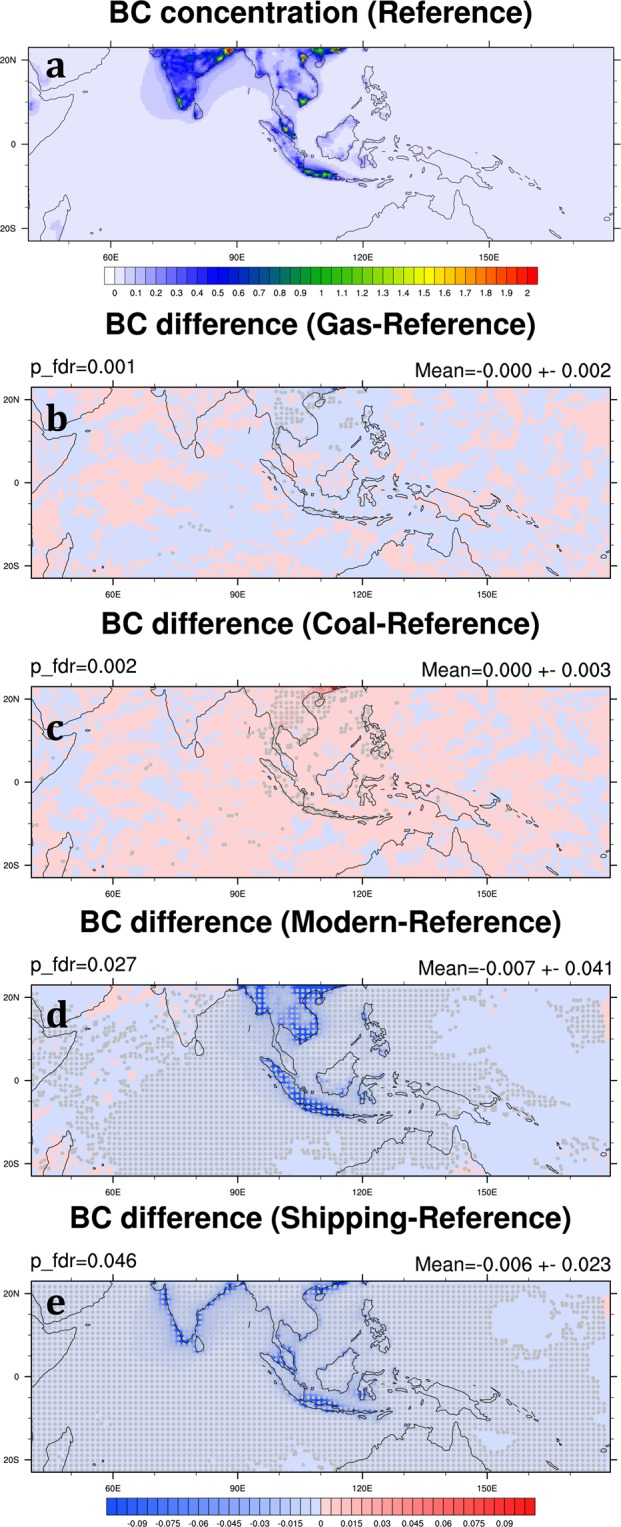
Same as Fig. 5 but for BC. These maps are generated by the NCAR Command Language (Version 6.4.0) [Software]. (2017). Boulder, Colorado: UCAR/NCAR/CISL/TDD. (http://dx.doi.org/10.5065/D6WD3XH5).

In the Reference Scenario, high monthly-mean concentrations of BC mainly occur both in the cities of Southeast Asia and widely across India (Fig. 6a). The mean concentration of BC in the SEA domain is 0.05 ± 0.00 μg m^−3^ (Table 3). In the Gas and Coal Scenarios, the change of BC concentration compared to the Reference Scenario is minor (Fig. 6b,c), less than 2% in the SEA domain (Table 4). However, BC concentration has a significant reduction in the Modern Scenario from its level in the Reference Scenario, especially in mainland Southeast Asia, Sumatra, and Java Island (Table 4; Fig. 6d). Substantial BC reductions in these major cities and their suburbs in the Modern Scenario, on the other hand, indicate high biofuel consumptions in these areas and the effectiveness in cutting BC concentration by replacing biofuel with natural gas in the residential sector. In other words, if the ASEAN governments want to regulate BC emissions, focusing on the residential sector would be more effective than focusing on the industry and power generation sectors. It is worth highlighting a relatively small BC reduction in the Malay Peninsula in the Modern Scenario compared with other major cities in Southeast Asia. This is because the primary BC emissions in this region come from road transportation; therefore, the changes of BC concentration in the Gas, Coal, and Modern Scenarios are all small. In the Shipping Scenario, most BC reductions happen in the coastal area, leading to a 21.6% decrease across the SEA domain (Table 4; Fig. 6e). Because diesel is widely used to fuel ocean-going ships, reducing shipping emissions can effectively reduce concentrations of several major pollutants.

**Table 4:** The mean change in percentage of Gas, Coal, Modern and Shipping Scenarios to Reference Scenario (i.e. (Gas-Reference)/Reference × 100%) for each species in the SEA domain (10°S–23°N in latitude, 90°E–150°E in longitude) averaged over 2006 and 2008.

	PM_2.5_	BC	OC	SO_4_	NO_3_	SO_2_	CO	O_3_
Gas - Ref.	−4.8 ± 0.7%	−1.0 ± 0.2%	0.4 ± 0.3%	−25.0 ± 5.4%	−4.1 ± 0.9%	−36.1 ± 1.2%	−0.1 ± 0.0%	−0.1 ± 0.1%
Coal − Ref.	8.6 ± 1.2%	1.7 ± 0.2%	−0.7 ± 0.4%	48.0 ± 10.3%	6.9 ± 1.6%	69.7 ± 2.4%	0.3 ± 0.1%	0.2 ± 0.1%
Modern − Ref.	−5.0 ± 0.6%	−41.7 ± 1.9%	−8.2 ± 1.8%	2.8 ± 0.9%	−0.5 ± 0.8%	4.1 ± 0.2%	−2.9 ± 0.3%	−0.1 ± 0.0%
Shipping − Ref.	−26.4 ± 3.4%	−21.6 ± 1.6%	−19.9 ± 1.6%	−27.7 ± 3.5%	−31.3 ± 5.4%	−27.3 ± 2.6%	−1.4 ± 0.2%	−12.9 ± 1.4%

**Figure 7:**
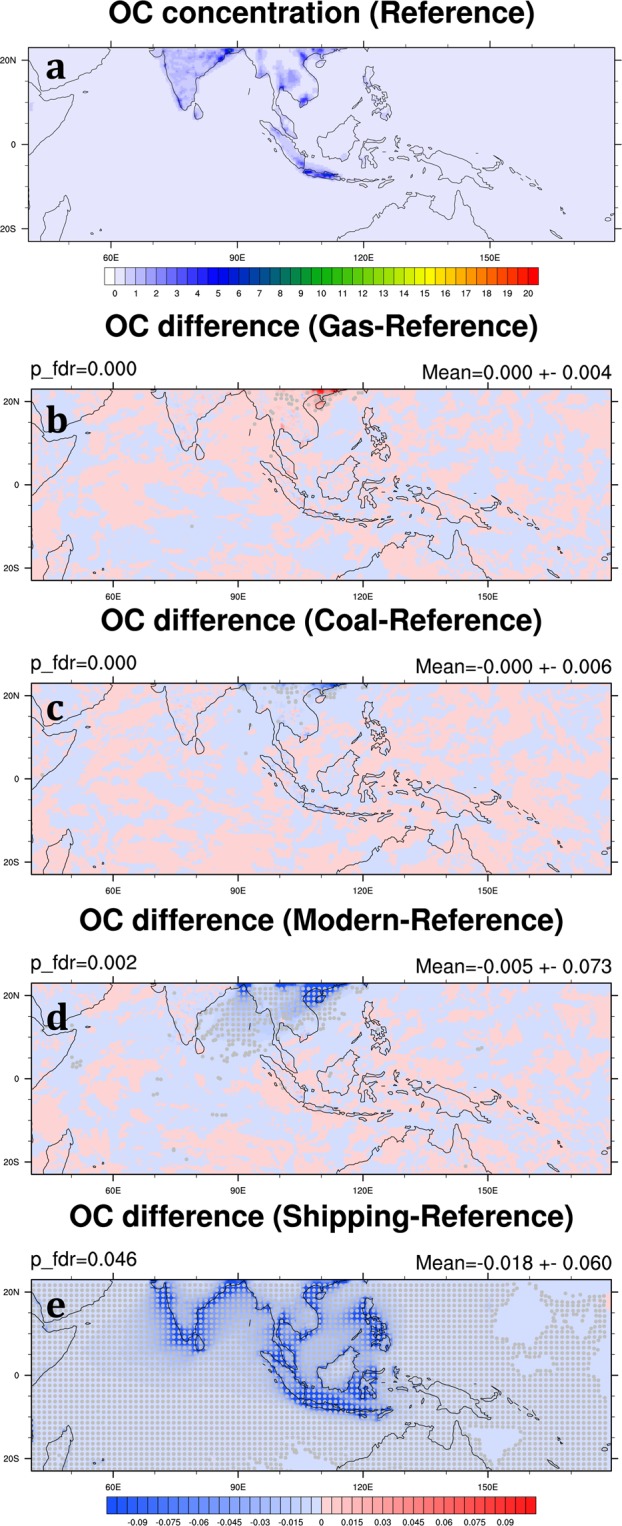
Same as Fig. 5 but for OC. These maps are generated by the NCAR Command Language (Version 6.4.0) [Software]. (2017). Boulder, Colorado: UCAR/NCAR/CISL/TDD. (http://dx.doi.org/10.5065/D6WD3XH5).

In the Reference Scenario, the monthly mean OC in the SEA domain is 0.19 ± 0.01 μg m^−3^ averaged over 2006 and 2008 (Table 3). High OC concentrations also mainly occur in the major cities in Southeast Asia and India (Fig. 7a). Compared with the Reference Scenario, the emission coefficient of OC in the industry sector is higher in the Gas Scenario while lower in the Coal Scenario (Table 2) so that OC concentration increases by 0.4% in the Gas Scenario and decreases by 0.7% in the Coal Scenario in the SEA domain (Table 4; Fig. 7b,c). This is because we refer the OC emission factor for “other” fuel combustion from Ohara, *et al*.^22^, which is higher than that of coal and oil combustion (Table 1). This setup also introduces higher OC emission in the Gas Scenario than the Reference Scenario (Fig. 3b). The spatial distribution of OC reduction in the Modern Scenario is similar to that in the Coal Scenario (Fig. 7d). However, the decrease in OC concentration in the Modern Scenario can be as high as 8.2% in the SEA domain, suggesting that reducing biofuel combustion in the residential sector is much more effective in reducing OC concentration in Southeast Asia than other emission mitigation measures in the power generation and industry sectors. The spatial distribution and percentage of OC decreases in the Shipping Scenario are similar to BC (Fig. 7e). The concentrations are reduced by 19.9% in the SEA domain (Table 4).

**Figure 8:**
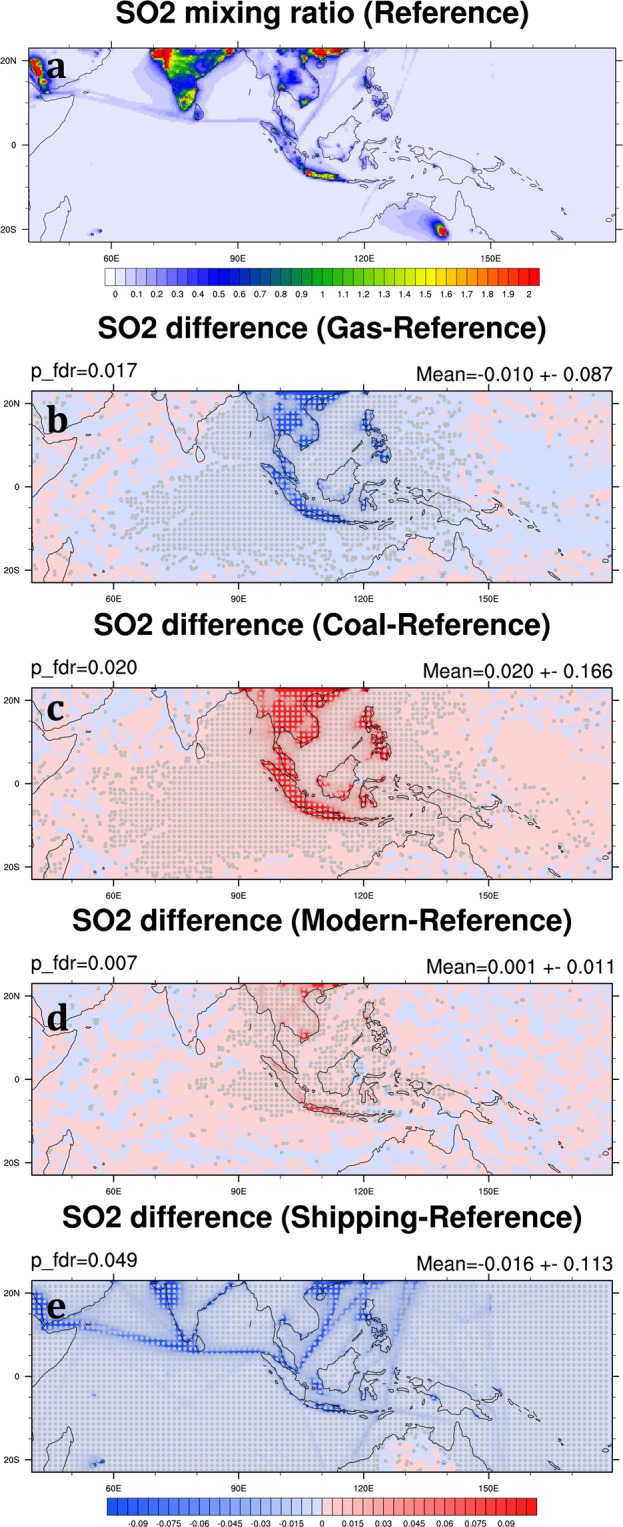
Same as Fig. 5 but for SO_2_ mixing ratio (ppbv). These maps are generated by the NCAR Command Language (Version 6.4.0) [Software]. (2017). Boulder, Colorado: UCAR/NCAR/CISL/TDD. (http://dx.doi.org/10.5065/D6WD3XH5).

**Figure 9:**
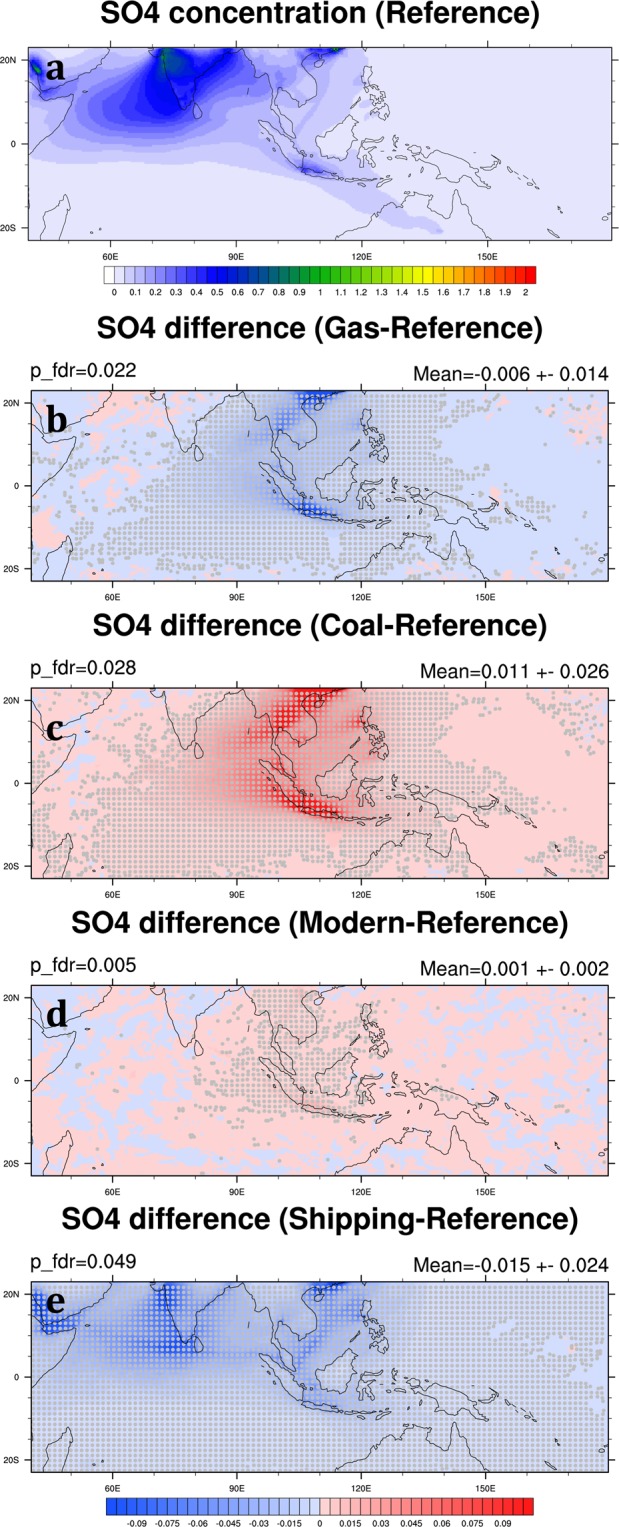
Same as Fig. 5 but for SO_4_. These maps are generated by the NCAR Command Language (Version 6.4.0) [Software]. (2017). Boulder, Colorado: UCAR/NCAR/CISL/TDD. (http://dx.doi.org/10.5065/D6WD3XH5).

SO_2_ is the predominant anthropogenic sulfur-containing air pollutant, and its oxidation produces sulfate. SO_2_ is primarily emitted from fossil fuel combustion in the power generation and industry sectors. Hence, high SO_2_ mixing ratio usually appears in high population areas and industrial cities, such as the northwest coast of India, Southern China, Bangkok, Manila, and Jakarta (Fig. 8a). In our simulations, high SO_4_ concentrations from the northwest coast of India and Southern China can even be transported downwind to the Arabian Sea and Bay of Bengal (Fig. 9a). In the Reference Scenario, the monthly mean SO_4_ is 0.06 ± 0.02 μg m^−3^ in the SEA domain averaged over 2006 and 2008 (Table 3). Both SO_2_ and SO_4_ have a significant reduction in the Gas Scenario by 36.1% and 25.0% in the SEA domain, respectively (Table 4; Figs 8b and 9b). Our result shows that the gas-favorite policy can effectively lower SO_2_ mixing ratio and SO_4_ concentration in the region. On the other hand, a coal-favorite policy can worsen air quality by elevating sulfate abundance. In the Coal Scenario, SO_2_ and SO_4_ in the SEA domain increase by 69.7% and 48.0%, respectively (Table 4). The impact areas of air quality due to sulfate particles are not only seen in the source but also in wider regions (Fig. 9c). Differing from the reduction of BC and OC in the Modern Scenario, SO_2_ mixing ratio increases in some major cities in Southeast Asia because natural gas combustion emits more sulfur-containing air pollutants than biofuel combustion. Therefore, the concentration of SO_4_ has been increasing by 2.8% in the SEA domain (Figs 8d and 9d). Our result in the Shipping Scenario shows removing shipping emission can effectively cut down SO_2_ emission. The reduction of SO_2_ mixing ratio happens not only at harbor areas but also along the shipping tracks, so does the reduction of SO_4_ concentration (Figs 8e and 9e). In the Shipping Scenario, SO_2_ and SO_4_ in the SEA domain decrease by 27.3% and 27.7%, respectively (Table 4).

**Figure 10:**
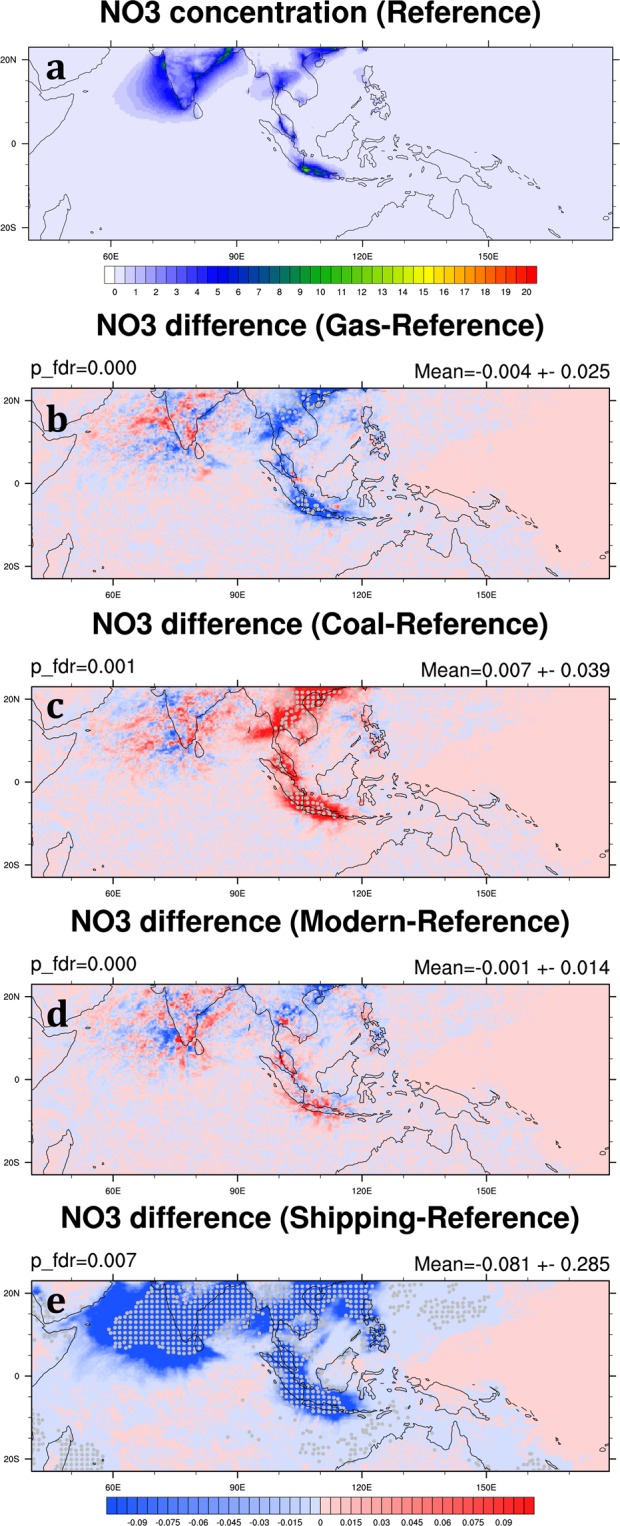
Same as Fig. 5 but for NO_3_. These maps are generated by the NCAR Command Language (Version 6.4.0) [Software]. (2017). Boulder, Colorado: UCAR/NCAR/CISL/TDD. (http://dx.doi.org/10.5065/D6WD3XH5).

NO_3_ aerosol is an important air pollutant and mainly produced from the reactions involving anthropogenic NO_x_ and O_3_. In the Reference Scenario, the monthly mean NO_3_ in the SEA domain is 0.32 ± 0.08 μg m^−3^ averaged over 2006 and 2008 (Table 3). High concentrations of NO_3_ mainly occur in Southern China, Thailand, the east coast of Malaysia, Java Island, and the offshore area of India (Fig. 10a). The main reduction zones in the Gas Scenario also correspond to the high NO_3_ concentration regions in the Reference Scenario, and the reduction of NO_3_ in the SEA domain is about 4.1% (Table 4; Fig. 10b). Compared with the growth of SO_4_ concentration in the Coal Scenario, the increase of NO_3_ is relatively small, only 6.9% in the SEA domain (Table 4). This is because that in our simulation, NO_3_ is the main component of PM_2.5_ and the substantial emissions of NO_x_ from road vehicles in the cities. Such a result explains the relatively minor changes of NO_3_ concentration in the Gas and Coal Scenarios compared with the changes of SO_4_. NO_3_ concentration does not have a significant change in the Modern Scenario except a decrease in southern China (Fig. 10d). Although the reduction of NO_3_ concentration is not substantial in the Gas and Modern Scenarios, it can be reduced by more than 30% in the Shipping Scenario in the SEA domain (Table 4). Furthermore, the reduction area covers not only ocean but also land (Fig. 10e).

**Figure 11:**
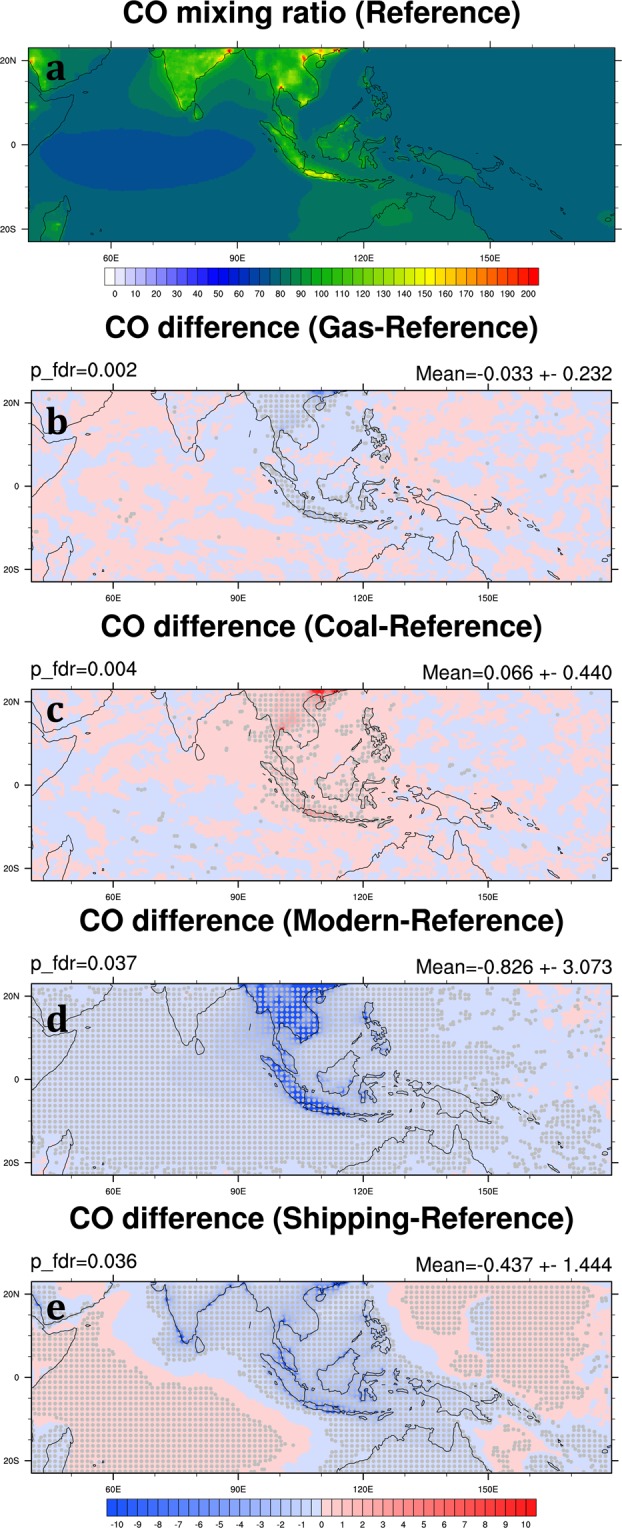
Same as Fig. 5 but for CO mixing ratio (ppbv). generated by the NCAR Command Language (Version 6.4.0) [Software]. (2017). Boulder, Colorado: UCAR/NCAR/CISL/TDD. (http://dx.doi.org/10.5065/D6WD3XH5).

In this study, we have also modified CO emissions based on different energy use scenarios (Fig. 3). CO is mainly from fossil fuel combustion, industrial processes, and biomass burning. In the Reference Scenario, high CO mixing ratio occurs mostly in the major cities in Southeast Asia and India, such as Hanoi, Bangkok, Jakarta, and Kolkata. The change of CO in the Gas and Coal Scenarios is relatively small, less than 0.4%; however, the change is substantial in the Modern Scenario (Table 4; Fig. 11b–d). As we mentioned above, CO comes from the combustion process, and biofuel combustion usually occurs under incomplete burning conditions. Therefore, replacing biofuel with natural gas can reduce CO mixing ratio by 2.9% in the SEA domain (Table 4). CO reduction also can be seen in the coastal regions in the Shipping Scenario, but the decreases over the open ocean are small (Fig. 11e).

**Figure 12:**
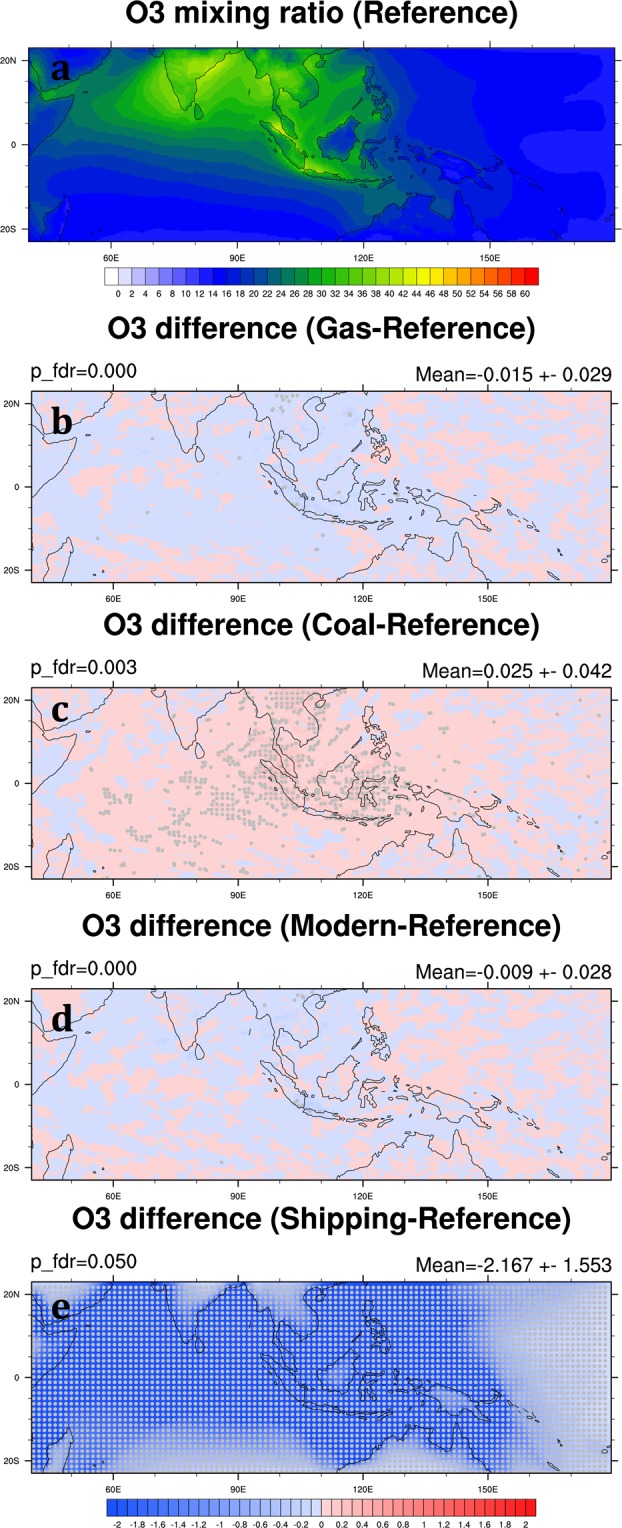
Same as Fig. 5 but for O_3_ mixing ratio (ppbv). These maps are generated by the NCAR Command Language (Version 6.4.0) [Software]. (2017). Boulder, Colorado: UCAR/NCAR/CISL/TDD. (http://dx.doi.org/10.5065/D6WD3XH5).

O_3_ production is mainly from photochemical reactions of precursors such as nitrogen oxides, volatile organic compounds, and CO, all mainly from anthropogenic emissions. A high level of near-surface O_3_ can cause public health issues^9^. In the Reference Scenario, the monthly mean O_3_ mixing ratio in the SEA domain is 23.3 ± 0.9 ppbv averaged over 2006 and 2008. The spatial distribution of high O_3_ mixing ratio is similar to CO (Fig. 12). The change of O_3_ in each scenario is rather minor, less than 0.3%, owing to the small change of precursors as well (Table 4; Fig. 12b–d). However, the reduction of O_3_ mixing ratio in the Shipping Scenario is substantial, −12.9% in the SEA domain (Table 4). It is due to the reduction of the major precursor nitrogen oxides in the region.

### Air quality impacts in five selected ASEAN cities under different fuel consumption scenarios

Population in Southeast Asia was about 634 million in 2015, with more than 100 million living in the major cities^6^. Owing to different industrial and urban infrastructures, air pollution sources are quite different from city to city. For those cities highly dependent on manufacturing as well as trading (in particular via shipping), our study provides a useful reference for making air pollution mitigation policies regarding future fuel consumption. In this section, we focus on five selected cities across Southeast Asia: Jakarta (Indonesia), Bangkok (Thailand), Hanoi (Vietnam), Singapore (Singapore), and Kuala Lumpur (Malaysia) (ordered according to population), and discuss their current air pollution issues and potential air quality adjustment under different hypothetical fuel consumption policies.

Jakarta is the largest capital city in Southeast Asia, with a population exceeding 10 million. The emissions are mainly from the residential, transportation, and industry sectors^1^. The mean PM_2.5_ concentration in the Reference Scenario is 14.7 ± 4.7 μg m^−3^ and about 60% of that is nitrate. The precursor of NO_3_, NO_x_, is also a major pollutant in Jakarta and causes severe smog and acid rain problems. In the Gas and Coal Scenarios, the change of NO_3_ concentration is about −4.5% and 7.2%, respectively. However, the most substantial changes in the aerosol composition come from SO_4_, −46.3% in the Gas Scenario and 85.6% in the Coal Scenario (Table 5). On the other hand, because BC and OC in Jakarta are not primarily emitted from industry and power generation, their changes in the Gas and Coal Scenarios are hence relatively small. Overall, the PM_2.5_ concentration decreases by 4.7% in the Gas Scenario and 8.0% in the Coal Scenario (Table 6). In the Modern Scenario, BC reduction can reach 56.9%, however, the concentrations of NO_3_ and SO_4_ increase by 1.1% and 6.1%, respectively. All these changes in opposite directions somewhat cancel out each other. As a result, the PM_2.5_ concentration would only experience a slight decrease (<1%) in the end if natural gas were chosen to replace biofuel in the residential sector. In fact, we find that targeting shipping emissions can lead to a significant improvement regarding PM_2.5_ concentration in Jakarta. Our result shows that removing shipping emissions can reduce NO_3_ by 25.4%, SO_4_ by 15.9%, OC by 16.6%, BC by 15.1%, and PM_2.5_ by 23.2% (Table 5).

**Table 5:** The mean change in percentage of Gas, Coal, Modern and Shipping Scenarios to Reference scenario (i.e. (Gas-Reference)/Reference × 100%) for each species in Jakarta (Indonesia), Bangkok (Thailand), Hanoi (Vietnam), Singapore (Singapore), and Kuala Lumpur (Malaysia) averaged over 2006 and 2008.

	PM_2.5_	BC	OC	SO_4_	NO_3_	SO_2_	CO	O_3_
**Jakarta**								
Gas - Ref.	−4.7%	−0.9%	0.5%	−46.3%	−4.5%	−53.2%	−0.8%	−0.5%
Coal - Ref.	8.0%	2.8%	0.3%	85.6%	7.2%	105.4%	1.8%	−0.1%
Modern - Ref.	−0.7%	−56.9%	0.4%	6.1%	1.1%	5.5%	−17.3%	−0.6%
Shipping - Ref.	−23.2%	−15.1%	−16.6%	−15.9%	−25.4%	−1.3%	−7.2%	−12.1%
**Bangkok**								
Gas - Ref.	−0.7%	−2.9%	0.8%	−34.5%	0.3%	−52.1%	−1.3%	−0.4%
Coal - Ref.	7.4%	6.3%	0.0%	67.8%	8.1%	101.6%	2.9%	0.2%
Modern - Ref.	1.3%	−22.9%	−0.3%	2.3%	3.1%	2.4%	−17.4%	−0.6%
Shipping - Ref.	−10.8%	−8.5%	−7.3%	−22.1%	−12.0%	−12.7%	−5.0%	−5.1%
**Hanoi**								
Gas - Ref.	−3.2%	−1.2%	0.0%	−38.6%	−3.3%	−40.5%	−0.4%	−0.2%
Coal - Ref.	5.8%	1.2%	−0.6%	72.8%	6.4%	79.0%	1.0%	0.5%
Modern - Ref.	−10.9%	−70.5%	−5.7%	4.1%	−0.4%	9.8%	−35.2%	−0.5%
Shipping - Ref.	−10.7%	−2.4%	−3.7%	−16.3%	−16.5%	−2.9%	−2.1%	−4.2%
**Singapore**								
Gas - Ref.	−1.1%	1.5%	1.1%	−28.0%	0.2%	−34.9%	−0.1%	0.1%
Coal - Ref.	3.9%	−2.2%	−2.2%	54.8%	1.7%	66.5%	0.2%	0.5%
Modern - Ref.	−4.0%	−15.2%	−0.8%	4.4%	−4.3%	2.8%	−3.2%	0.2%
Shipping - Ref.	−68.7%	−18.8%	−21.5%	−42.0%	−78.7%	−51.1%	−4.1%	−21.9%
**Kuala Lumpur**								
Gas - Ref.	−1.6%	0.3%	0.3%	−30.7%	−2.5%	−37.7%	0.0%	0.2%
Coal - Ref.	4.7%	−0.5%	−0.5%	63.3%	7.6%	73.6%	0.5%	0.8%
Modern - Ref.	1.4%	0.3%	0.9%	5.0%	2.2%	4.1%	−4.2%	0.2%
Shipping - Ref.	−19.8%	−2.0%	−2.8%	−29.7%	−35.5%	−11.8%	−3.3%	−10.5%

**Table 6:** Same as Table 4 but for the simulations of Gas, Coal, and Modern Scenario with the emission modification in the whole model domain (referred to as Gas_ALL, Coal_ALL, and Modern_ALL, respectively).

	PM_2.5_	BC	OC	SO_4_	NO_3_	SO_2_	CO	O_3_
Gas_ALL - Ref.	−5.8 ± 1.8%	−1.0 ± 0.3%	0.6 ± 0.3%	−36.3 ± 4.0%	−3.6 ± 2.2%	−36.8 ± 1.0%	−0.2 ± 0.1%	−0.2 ± 0.4%
Coal_ALL - Ref.	11.4 ± 2.4%	1.7 ± 0.2%	−1.2 ± 0.4%	69.0 ± 6.1%	7.4 ± 1.7%	71.0 ± 2.1%	0.3 ± 0.1%	0.3 ± 0.0%
Modern_ALL - Ref.	−5.5 ± 0.8%	−43.3 ± 1.3%	−10.5 ± 2.0%	4.1 ± 0.9%	−0.5 ± 0.9%	4.2 ± 0.2%	−3.0 ± 0.3%	−0.1 ± 0.1%

In Bangkok, the monthly mean PM_2.5_ concentration in the Reference Scenario is 10.0 ± 3.6 μg m^−3^. NO_3_ and OC are the dominant components of PM_2.5_ with 50.4% and 25.7%, respectively. Besides BC, the relative change of each species in the Gas and Coal Scenarios in Bangkok is similar to that in Jakarta. BC decreases 2.9% in the Gas Scenario in Bangkok and increases 6.3% in the Coal Scenario. Similar to the case of Jakarta, the significant BC reduction comes from the Modern Scenario, by 22.9%; nevertheless, this still cannot compensate the increase of SO_4_ and NO_3_. Hence, PM_2.5_ concentration in Bangkok slightly increases in the Modern scenario (Table 5). Shipping emission is not the most crucial pollution source in Bangkok to total PM_2.5_ (only contributing about 10%); however, shipping is a substantial source for SO_4_ concentration (22.1%), and thus a good target for sulfate aerosols mitigation policies in Bangkok.

The primary emission sectors in Hanoi are road transportation and residential. The mean PM_2.5_ concentration in Hanoi in the Reference Scenario is 9.8 ± 1.7 μg m^−3^. NO_3_ and OC are still the dominant components of PM_2.5_ with 39.7% and 29.4%, respectively. However, the proportion of BC to PM_2.5_ in Hanoi is 12.8%, 4~5 times higher than the BC percentage in Jakarta or Bangkok. Because the major pollutants do not come from industry and power generation, the changes of various aerosol species in the Gas or Coal Scenario in Hanoi are relatively small (Table 5). In the Modern Scenario, however, the massive hypothetical reduction in biofuel combustion would reduce BC concentration by 70.5%, and OC by 5.7%. It could result in a significant improvement of PM_2.5_ concentration in Hanoi by reducing it nearly 11%. We also would like to highlight an abundant CO reduction, about 35% in the Modern Scenario. All these results suggest that replacing biofuel by natural gas as a combustion fuel in the residential sector in Hanoi could efficiently reduce PM_2.5_ concentration by 11% and CO mixing ratio by 35%. In addition, shipping emissions also play an important role in causing air pollution in Hanoi. Removing shipping emissions in the Shipping Scenario lowers NO_3_ and SO_4_ concentrations by 16.5% and 16.3%, respectively, and PM_2.5_ by 10.7%, comparable to the effect of the Modern Scenario (Table 5).

Singapore has an active and dynamic industrial sector including chemical and metallurgic industries as well as a major petroleum-refining center in Southeast Asia. Additionally, power plants generate electricity by burning natural gas and, to a lesser extent, oil. Waste incinerators, busy seaport traffic, and a major airport in the region also make the emission map more complicated^29^. In the Reference Scenario, the mean PM_2.5_ concentration in Singapore is 2.5 ± 2.4 μg m^−3^ averaged over 2006 and 2008, and 60.9% of PM_2.5_ comes from nitrate aerosols. The most substantial change in aerosol composition in the Gas and Coal Scenarios occurs to SO_4_ with −28.0% and 54.8%, respectively. Based on the report published by Energy Market Authority, the proportion of natural gas burning in Singapore’s power generation is 95.2%^30^, much higher than other countries in Southeast Asia. Pollutants generated by coal burning in Singapore hence come mainly from transboundary atmospheric transport. On the other hand, shipping emission is a large pollution source in Singapore, though the government has not paid much attention to it until recently. In 2017, the International Maritime Organization (IMO) has set a 0.5% global cap on SO_2_ content in marine fuels, a significant reduction from the current limit of 3.5%, in order to achieve the 2020 target of reducing air pollutions due to international shipping^31^. In our simulation, at about 78.7%, shipping emissions make the most significant contribution to NO_3_ concentration in Singapore. Besides that, removing shipping emissions also reduces SO_2_ mixing ratio dramatically by 51.1%, SO_4_ concentration by 42.0%, and overall PM_2.5_ concentration by 68.7%. We also see a substantial reduction of O_3_ as high as 21.9% in the Shipping Scenario (Table 5).

Kuala Lumpur is a city of 1.76 million people in Southeast Asia, with an annual mean PM_2.5_ concentration derived in the Reference Scenario of 6.8 ± 3.2 μg m^−3^. Compared with the other cities discussed previously, the proportion of BC and OC to PM_2.5_ concentration is high in Kuala Lumpur, at 18.6%, and 25.5%, respectively. On the other hand, the proportion of NO_3_ component is relatively low, only 38.2%. Somewhat uniquely, the concentration of BC and OC are barely changed in the Gas, Coal, and Modern Scenarios compared to the Reference Scenario (Table 5). This indicates that the major emission source of BC and OC is road transportation, not the industry, power generation, or residential sectors. Shipping emissions have a significant contribution to NO_3_ and SO_4_ by 35.5% and 29.7%, respectively. Furthermore, as demonstrated in the Shipping Scenario, reducing shipping emissions could result in a 19.8% reduction in PM_2.5_ concentration, and this appears to be the most effective target for air quality improvement than other energy use scenarios.

### A discussion of the sensitivity of chemical concentration in Southeast Asia to transboundary pollution

The considerably high concentration of air pollution in India within the model domain makes it hard to overlook its potential impact on air quality in Southeast Asia. Hence, we have estimated the impact of transboundary pollution from India on each chemical concentration in Southeast Asia under different hypothetical fuel consumption scenarios. This has been done by comparing the results from simulations with emission modifications applied only to the Southeast Asia subdomain with results from simulations with emission modifications applied to the who model domain, including the South Asia subdomain (referred to as _ALL below).

Compared with the reduction of PM_2.5_ concentration in the Gas Scenario, the reduction of PM_2.5_ concentration in Gas_ALL (the Gas Scenario with emission modification in the whole model domain) is 1.0% more, which mainly comes from the reduction of SO_4_ by 11.3% (25.0% in Table 4 versus 36.3% in Table 6). In other words, if all countries in the model domain would use gas to replace coal in the power generation and industry sectors, it could further reduce SO_4_ concentration by 11.3% in Southeast Asia (from −25.0% to −36.3%). On the other hand, the substantial increases of SO_4_ and PM_2.5_ concentration in Coal_ALL are expected based on the high SO_4_ concentration observed in India in the Reference Scenario (Fig. 9a). If the coal-favorite policy in industry and power generation were also applied in India, it could worsen air quality not only in India locally but also neighboring countries in Southeast Asia. Compared with the Coal Scenario, Coal_ALL indeed adds an additional 21% of SO_4_ in Southeast Asia (in terms of increase from the Reference Scenario, 48.0% in Table 4 versus 69.0% in Tables 6), and 2.8% in PM_2.5_ (from 8.6% to 11.4%). Our results show that the simulations with the gas-favorite and coal-favorite emission modifications in the whole domain do not change the concentrations of chemical species other than SO_4_ significantly in Southeast Asia (<1%), implying that the abundance of these species in Southeast Asia is less affected by transboundary pollution.

High BC and OC concentration in India are also observed in the Reference Scenario (Figs 6a and 7a). As discussed previously, BC and OC are both reduced significantly in the Modern Scenario. We find that by reducing biofuel consumption and using natural gas and electricity in the residential sector in both Southeast and South Asia, BC and OC abundances in the SEA domain can be reduced 43.3% and 10.5%, respectively (Table 6). On the other hand, compared with the Modern Scenario with the Modern_ALL Scenario shows that the major contributors of BC and OC concentration in Southeast Asia are primarily the local sources: transboundary BC and OC only affect 1.6% (from 41.7% to 43.3%) and 2.3% (from 8.2% to 10.5%) of BC and OC concentration in the SEA domain, respectively.

### Summary and conclusions

In this study, we have designed five hypothetical fuel consumption scenarios: *the Reference*, *Gas*, *Coal*, *Modern, and Shipping Scenarios*. By applying pollutant emissions resulting from these scenarios in a regional weather-atmospheric chemistry model, we have examined the outcomes in air quality over Southeast Asia in response to different fuel usage policies in the power generation, industry, or residential sector. Through the analyses based on the comparison of the results from these scenarios, we demonstrate that a practice favoring the dominant usage of coal in the energy mix (*the Coal Scenario*) could lead to a significant increase in the aerosol and gas emissions and a worsening of air quality in the region. On the other hand, a practice of shifting fuels in the energy mix from coal to natural gas (*the Gas Scenario*) would effectively lower the abundance of air pollutants. Besides potential policies regarding the fuel consumptions in the industrial and energy generation sectors, we have also investigated the consequences in air quality by either reducing the use of traditional biofuels in the residential sector (*the Modern Scenario*) or the emissions from shipping (*the Shipping Scenario*).

Sulfate aerosols (SO_4_) are the primary pollutant produced by coal burning in the power generation and industry sectors. In the Gas Scenario, the reduction of SO_4_ concentration can reach 25% in Southeast Asia by shifting fuels in energy mix from coal to natural gas, resulting in a decrease in PM_2.5_ concentration by 4.8% compared to the Reference Scenario. On the other hand, in the Coal Scenario, shifting fuels from natural gas to coal in the energy mix could make air quality much worse by increasing SO_4_ concentration by 48% and PM_2.5_ concentration by 8.6%. In the Modern Scenario, substantial reduction of BC and OC concentration by 41.7% and 8.2%, respectively, in Southeast Asia, results in a 5.0% decrease of PM_2.5_ concentration, even more than the reduction of PM_2.5_ in the Gas Scenario. This result shows that people in Southeast Asia still commonly use biofuel in their daily activities. Specifically, based on our analyses, shipping emission affects air quality in Southeast Asia significantly, responsible for more than 26% of PM_2.5_ concentration. Our results suggest that mitigation policies targeting total PM_2.5_ concentration in Southeast Asia should focus not only on the coal and biofuel consumption sectors but also on shipping emissions.

We find the impacts of various policies on key pollutants vary across 5 selected cities. In the Gas Scenario, all five cities show a substantial reduction in sulfate aerosols varying from 28.0% to 46.3%. Jakarta and Bangkok can reduce even more than half of SO_2_ mixing ratio in the Gas Scenario. However, if the regulation policy aims to target BC concentration, replacing biofuel by natural gas in the residential sector can reduce BC concentration in Jakarta and Hanoi more substantially than others by 57.0% and 70.6%, respectively. On the other hand, shipping emission has a significant impact on the air quality in the selected cities, especially in Singapore. By eliminating shipping emissions, the PM_2.5_ concentration in Singapore can be reduced by 68.7%. We also see that the substantial reduction of O_3_ mixing ratio in the Shipping Scenario can be as high as 21.9% in Singapore. In our study, we did not include road transportation in Southeast Asia in making our hypothetical scenarios. Nevertheless, our results actually imply the potential importance of road transportation in mitigating PM_2.5_ in several major cities in the region. Future studies should directly address the roles of various policies regarding road transportation sector in the mitigation of air pollution for Southeast Asia.

We have investigated the impact of transboundary pollution from outside of Southeast Asia on air quality in Southeast Asia by designing another set of the Gas, Coal, and Modern Scenarios with emission modification applied to the entire model domain including South Asia. Our results show that SO_4_ concentration in Southeast Asia can be influenced by 11.3% by transboundary pollution. Whereas, all other key pollutants are much dependent on local emissions, transboundary transport only accounts less than 1% of their changes.

## Supplementary information

Supplement of The Impact of Future Fuel Consumption on Regional Air Quality in Southeast Asia
