# Supplementary material for: The Impact of Future Fuel Consumption on Regional Air Quality in Southeast Asia
Source: Sci Rep. 2019 Feb 25;9:2648. doi: 10.1038/s41598-019-39131-3 (PMC6389970; doi:10.1038/s41598-019-39131-3)
Supplement: Supplementary file 1 — Supplement of The Impact of Future Fuel Consumption on Regional Air Quality in Southeast Asia [file 41598_2019_39131_MOESM1_ESM.pdf]

Supplement of

The Impact of Future Fuel Consumption on Regional Air Quality in Southeast Asia

Hsiang-He Lee[1@], Oussama Iraqui[2], and Chien Wang[1,3]

[1] Center for Environmental Sensing and Modeling, Singapore-MIT Alliance for Research and Technology, Singapore

[2]Energy and Environmental Engineering Department, National Institute of Applied Science of Lyon (INSA Lyon), France

[3] Center for Global Change Science, Massachusetts Institute of Technology, Cambridge, MA, U.S.A.

Submitted to

Scientific Reports

July 2, 2018

[@]Corresponding author address: Dr. Hsiang-He Lee, 1 CREATE Way, #09-03 CREATE Tower, Singapore, 138602

E-mail: hsiang-he@smart.mit.edu

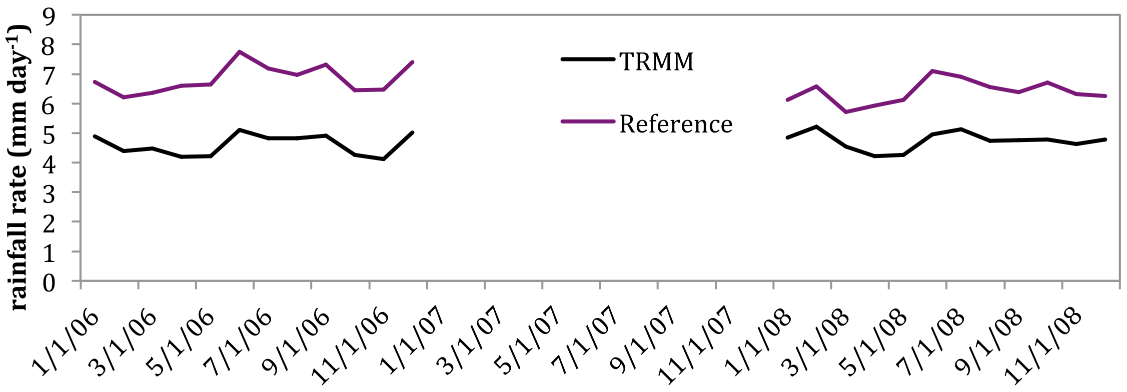

Figure S1. Time series of domain-averaged monthly precipitation rate from TRMM dataset and the Reference Scenario in the year of 2006 and 2008. The figure is generated by the Microsoft Excel (Version 14.7.2).

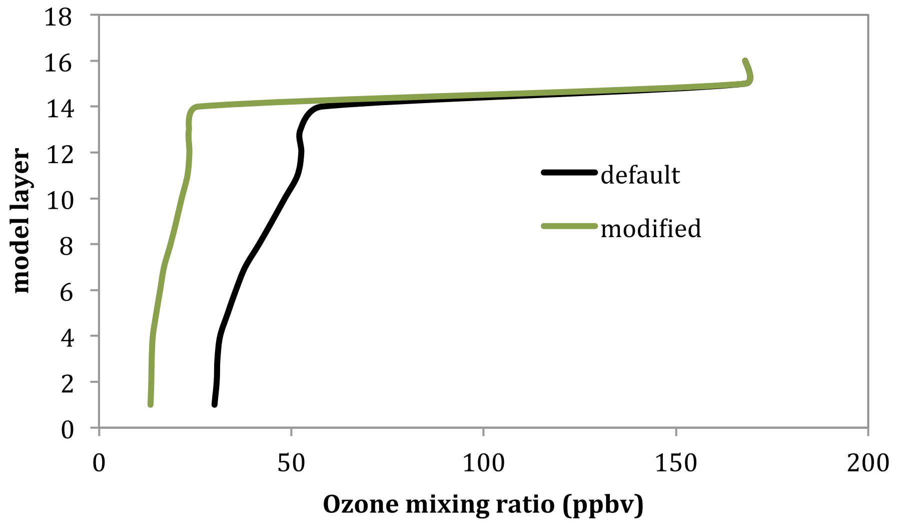

Figure S2. Default and modified ozone profile using in the lateral boundary condition of WRF-Chem. The figure is generated by the Microsoft Excel (Version 14.7.2).

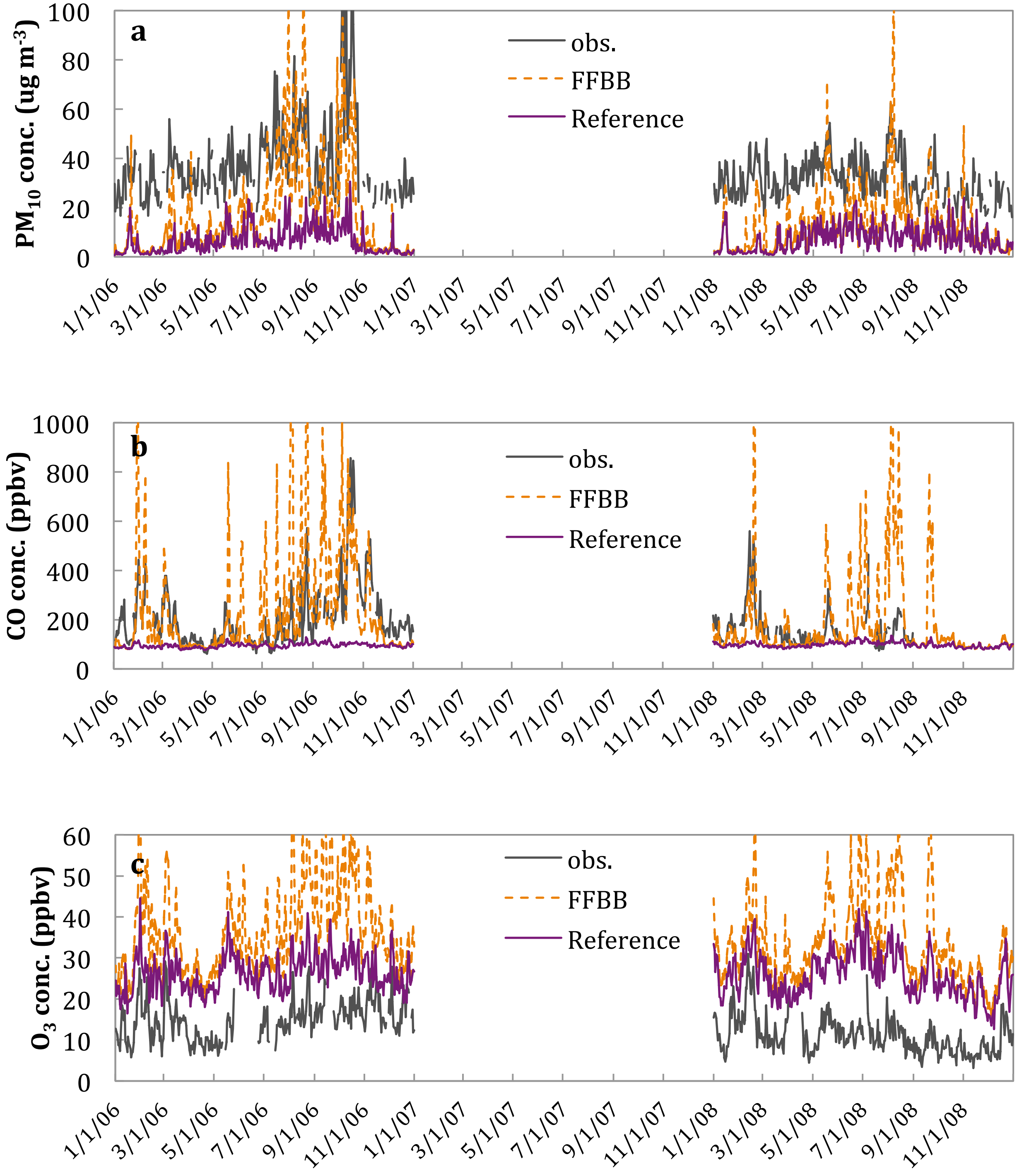

Figure S3. (a) Time series of daily surface PM_{10} ($\mu g\ m^{-3}$; AQI derived) from the ground-based observations, both fossil fuel and biomass burning emissions simulated results (FFBB; from Lee et al.[1]), and fossil fuel emissions only simulated results (Reference; from the Reference Scenario this study) in Kuala Lumpur, Malaysia in the year of 2006 and 2008. (b) Time series of daily surface CO mixing ratio (ppbv) from the ground-based observations (obs.), both fossil fuel and biomass burning emissions simulated results (FFBB; from Lee et

al.[1]), and fossil fuel emissions only simulated results (Reference; from the Reference Scenario this study) in Bukit Kototabang, Indonesia in the year of 2006 and 2008. (c) Same as (b) but surface O_3. These figures are generated by the Microsoft Excel (Version 14.7.2).

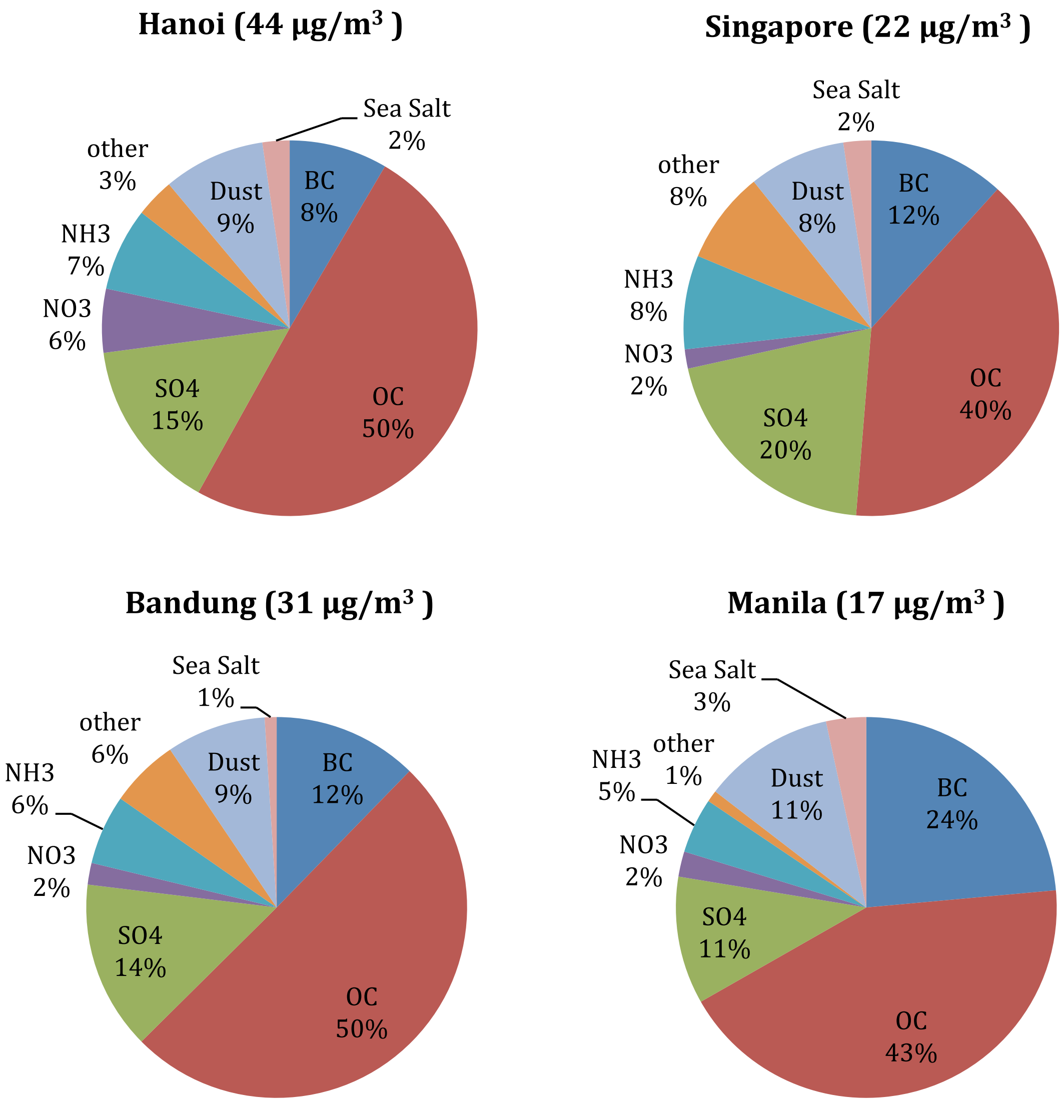

Figure S4. Pie chart of chemical components of $PM_{2.5}$ from the Surface PARTiculate mAtter Network (SPARTAN) filter samples in Hanoi (Vietnam), Singapore (Singapore), Bandung (Indonesia), and Manila (Philippines). These figures are generated by the Microsoft Excel (Version 14.7.2).

References:

1 Lee, H. H. *et al.* Impacts of air pollutants from fire and non-fire emissions on the regional air quality in Southeast Asia. *Atmos. Chem. Phys.* **18**, 6141-6156, doi:10.5194/acp-18-6141-2018 (2018).
